# Uses of Antipyrin and Antifebrin

**Published:** 1888-01

**Authors:** 


### *USES OF ANTIPYRIN AND ANTIFEBRIN.*

Demieville confirms the observations of other clinicians regarding the usefulness of antipyrin in neuralgia. The dose is seven grains, three times daily. Pain disappears frequently immediately after ingestion of the drug, and, in most instances, within one-half to one hour. Sleep is also induced by the remedy. The system becomes accustomed to antipyrin only after a prolonged use, and a discontinuation of a few days suffices to re-establish the activity of the drug. Thus far, sciatica, lumbago, and a few other forms of neuralgia were treated satisfactorily with antipyrin. Dujardin-Beaumetz recommends antifebrin as an anodyne in neuralgic or rheumatic pain. He asserts that the remedy acts even in such cases of pain as are based upon an alteration or compression of nerves (neuritis optica); likewise, in pain referring to a multiple sclerosis. In epilepsy, antifebrin showed also a favorable action. The dose is twenty to thirty grains three to four times daily. Francotte found that antifebrin reduces the reflex activity, while antifebrin increases the same. Both produce tonic and clonic cramps. Hence, the two drugs should be exhibited in different nervous diseases. In tabes, antipyrin proves useful in checking the lancinating pains, though the ataxia increases during the use of the drug; this disadvantage is removed by discontinuing the remedy. In neuralgia of the head (as Faust also proved), antipyrin is by far the most effectual remedial and palliative agent. It is best given in capsules containing seven to fifteen grains each.—*Annales de la Societe Medico-Chir. de Liege.*

